# Supplementary material for: The First Transcriptomic Atlas of the Adult Lacrimal Gland Reveals Epithelial Complexity and Identifies Novel Progenitor Cells in Mice
Source: Cells. 2023 May 21;12(10):1435. doi: 10.3390/cells12101435 (PMC10216974; doi:10.3390/cells12101435)
Supplement: Supplementary file 1 [file cells-12-01435-s001.zip › cells-2380877-supplementary figures.pdf]

Supplementary materials for:

The First Transcriptomic Atlas of the Adult Lacrimal Gland Reveals Epithelial Complexity and Identifies Novel Progenitor Cells in Mice

Vanessa Delcroix [1], Olivier Mauduit [1], Hyun Soo Lee [1,2], Anastasiia Ivanova [1], Takeshi Umazume [1], Sarah M. Knox [3,4], Cintia S. de Paiva [5], Darlene A. Dartt [6] and Helen P. Makarenkova [1,*]

Supplementary figures

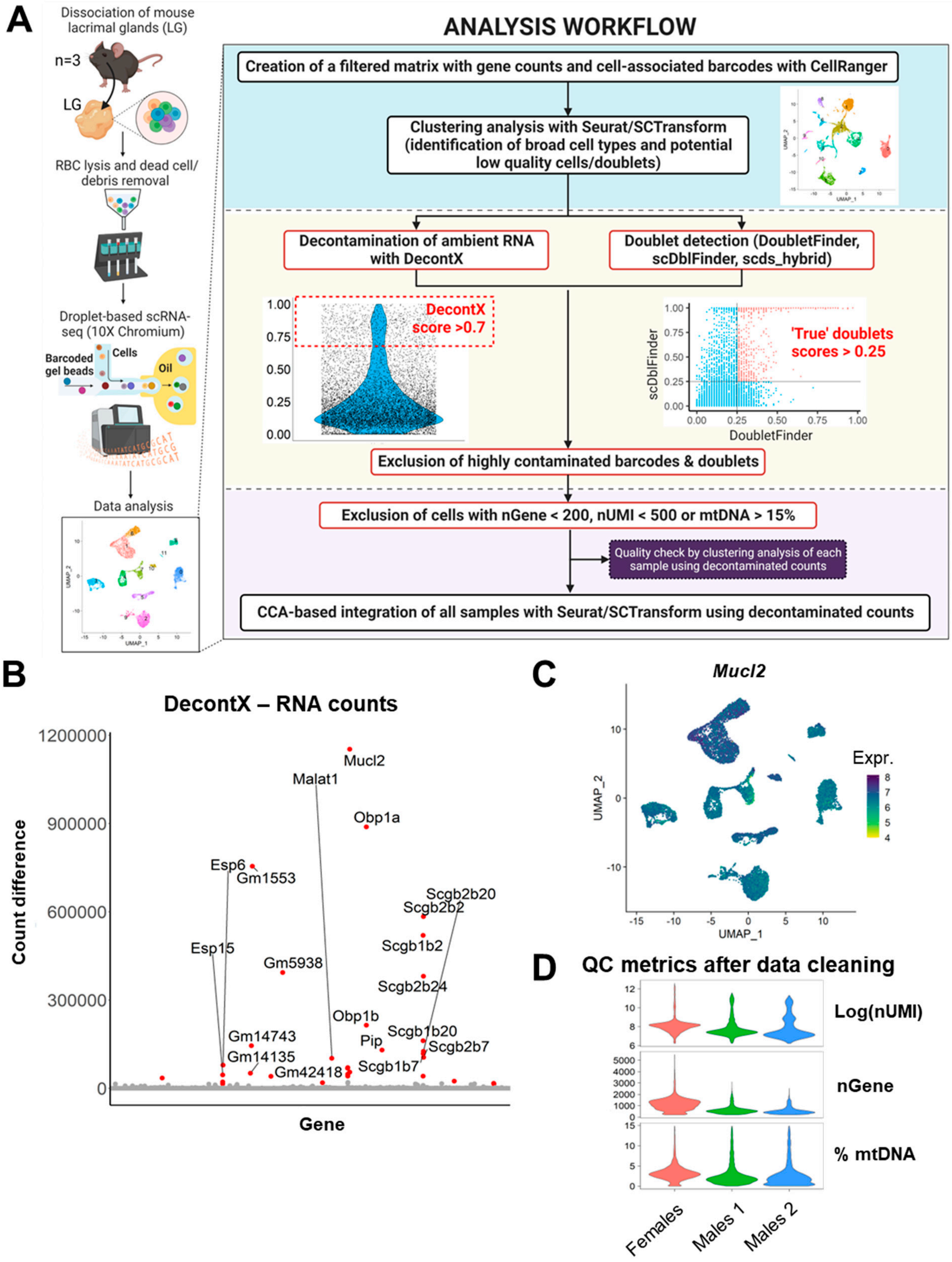

Figure S1. Analysis workflow and quality control for scRNAseq data from adult mouse lacrimal gland. (**A**) Schematics representing the different steps for the analysis of mouse LG by scRNAseq. For each sample, the lacrimal glands (LG) from 3 mice were harvested and dissociated into single cells. Erythrocytes and dead cells/debris were depleted from the cell suspension before loading onto the microfluidic chip for analysis with the Chromium system from 10X Genomics. Sequencing data were processed with CellRanger (10X Genomics) and the resulting filtered matrix containing gene counts and cell-associated barcodes was

analyzed using the R package Seurat. Broad cell annotations were used for analysis with the SingleCellTK R package including celda/DecontX (to reduce ambient RNA contamination) and doublet detection packages (DoubletFinder, scDblFinder, scds_hybrid). Cells with a high DecontX score (> 0.7) and 'true doublets' (DoubletFinder/scDblFinder joint scores > 0.25) were excluded from downstream analysis. Following high resolution clustering using decontX-corrected counts, cells associated with low quality metrics (nGene < 200, nUMI < 500, or mtDNA > 15%) were excluded. Then, integration of samples and clustering analysis was done using SCTransform-based normalization of DecontX-corrected counts, following Seurat's recommended workflow. Scheme created with BioRender (www.biorender.com) (**B**) This graph shows the difference in counts between the RNA and DecontX assays (containing uncorrected and corrected counts, respectively) for a given gene. Most corrected genes (red dots) correspond to acinar transcripts. (**C**) UMAP plot for *Mucl2* expression in the whole LG atlas showing ubiquitous detection of *Mucl2* transcript. (**D**) Distribution of the final number of RNA molecules (nUMI, log scale), genes (nGene) and percent of mitochondrial genes (% mtDNA) across samples after filtering out low quality cells and doublets.

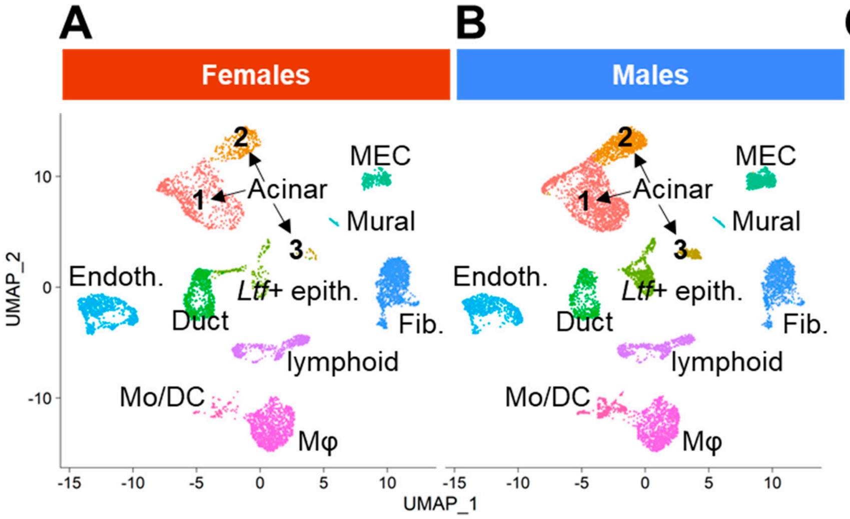

C

Cluster %	Females	Males
Acinar 1	10.7	20.1
Acinar 2	4.1	10.6
Acinar 3	0.3	3.2
Ltf+ epith.	3.0	8.0
Duct	12.7	6.1
MEC	4.5	6.1
Mural	0.6	0.8
Endothelial	12.2	7.9
Fibroblast	22.1	12.4
Mφ	18.6	12.9
Mo/DC	1.1	5.2
Lymphoid	10.1	6.7

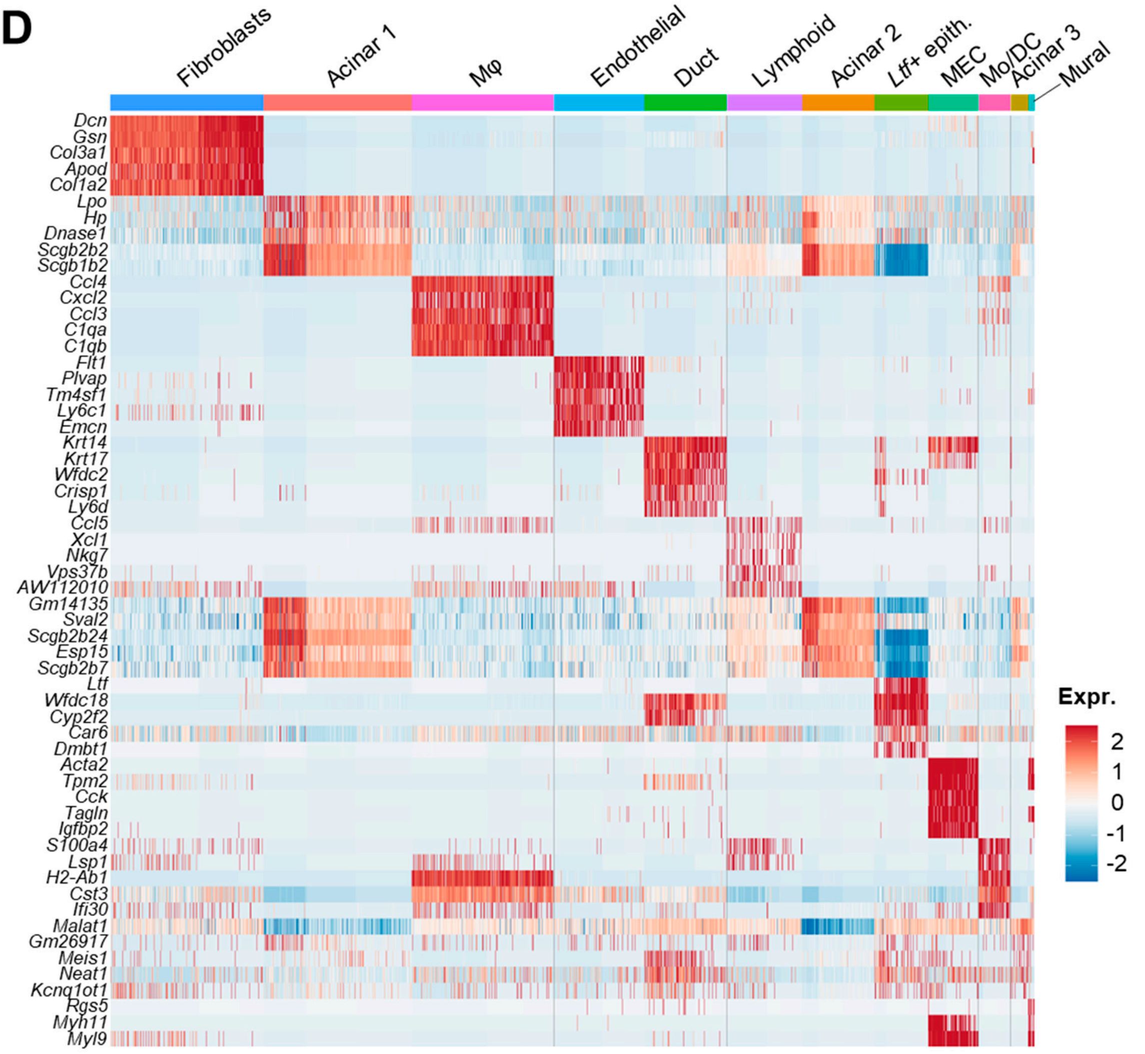

Figure S2. Clustering analysis of the two months old mouse lacrimal gland.

(**A-B**) Data from all three samples (one female, two males, 9 mice in total) were integrated and UMAP plot was split to compare cell composition in (**A**) females and (**B**) males. Cells were colored based on cluster identity. (**C**) Table summarizing the proportion of each cluster in the LG, respective to mouse sex. (**D**) Gene expression heatmap of the top-5 markers that are most conserved across

samples for the main cell types composing the LG. Color scale corresponds to the scaled average expression level (red: high; blue: low) and the upper bar is colored by cluster identity.

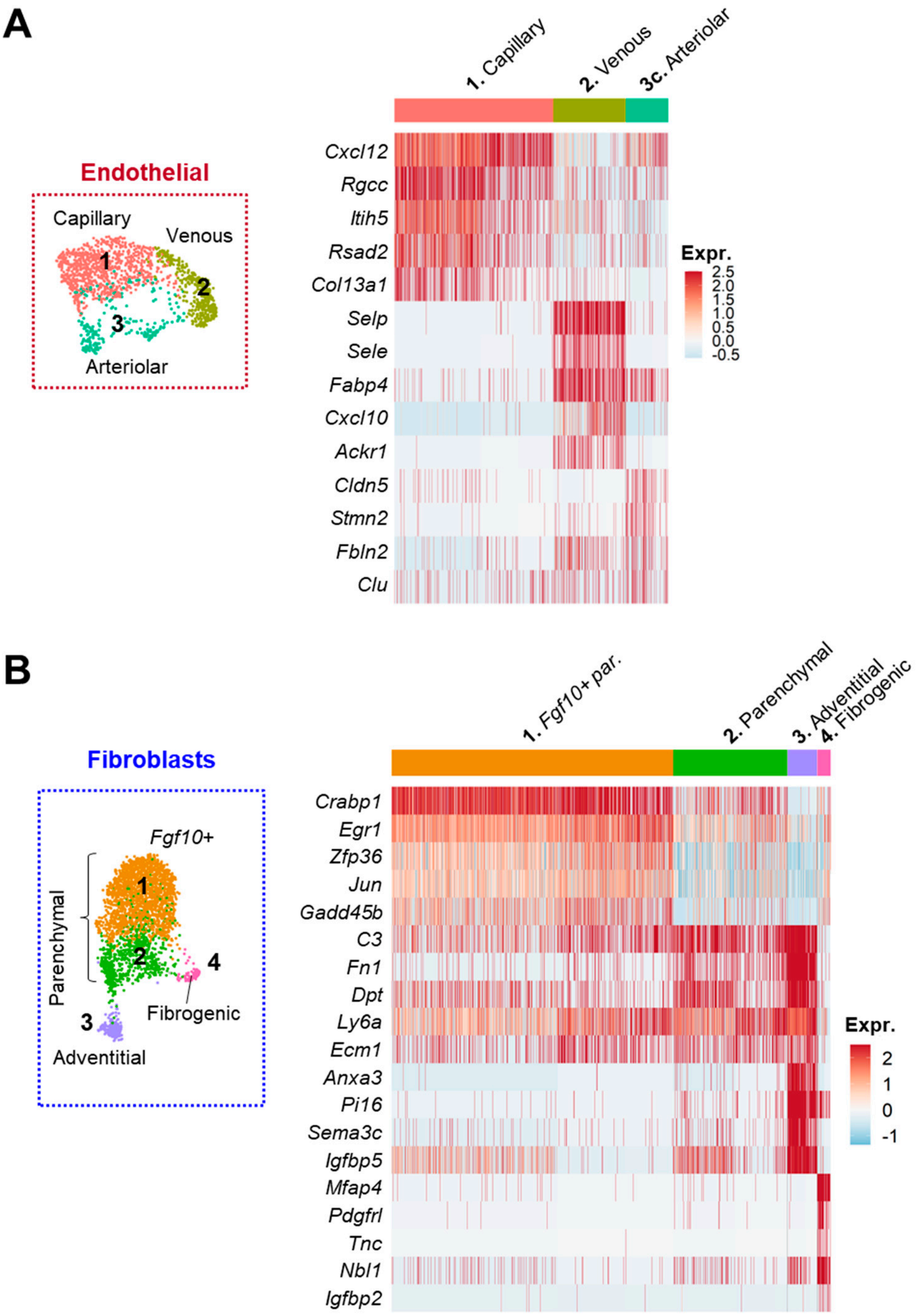

Figure S3. Subclustering analysis of the stromal populations composing the mouse lacrimal gland.

(**A-B**) Gene expression heatmaps of the top-5 markers for (**A**) endothelial and (**B**) fibroblasts subclusters. UMAP plots on the left-hand side were cropped from the whole LG atlas to visualize the repartition of the subclusters. Clusters are colored by identity. Color gradient on the heatmap corresponds to the scaled average expression level (red: high; blue: low) and the upper bar is colored by cluster identity.

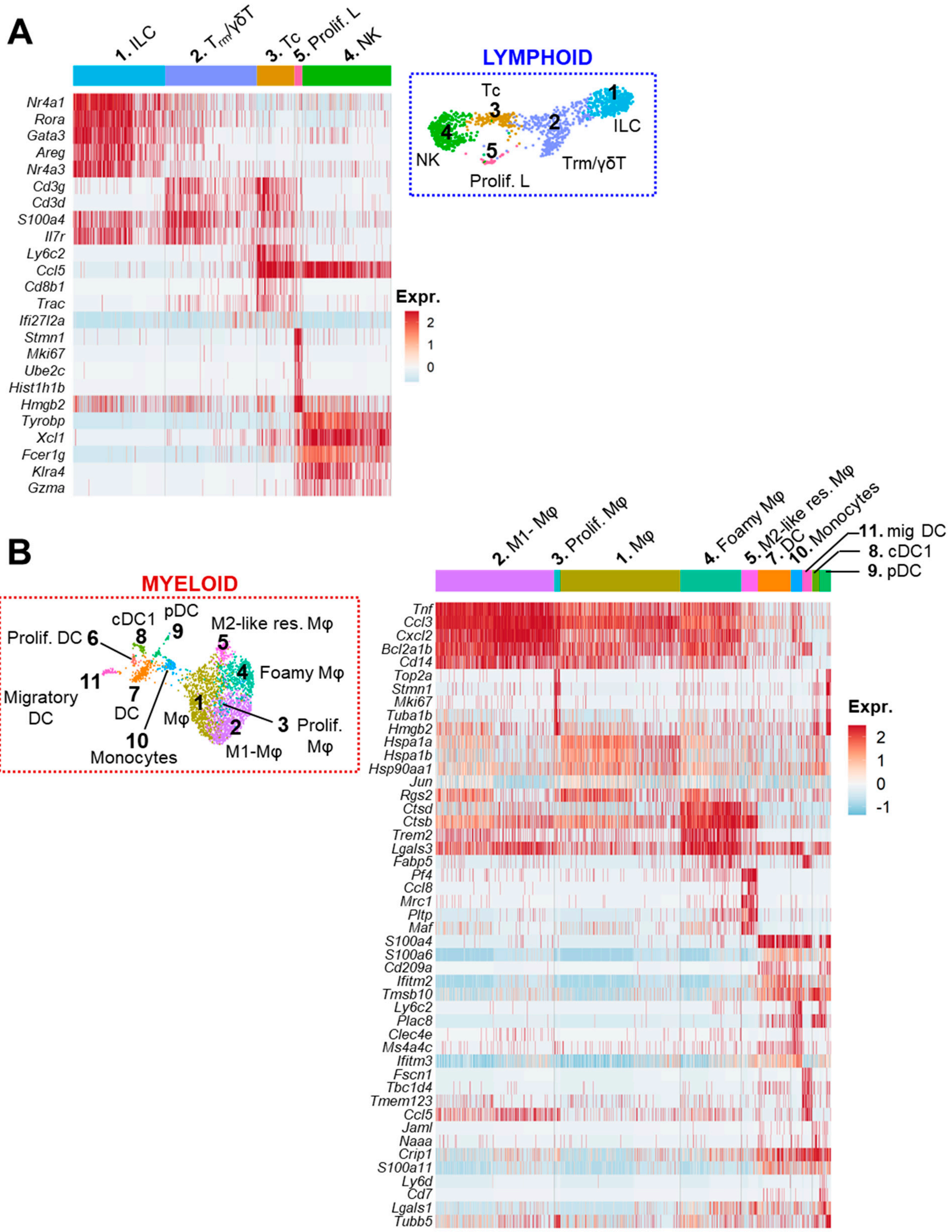

Figure S4. Subclustering analysis of the immune cell populations composing the mouse lacrimal gland.

(**A-B**) Gene expression heatmaps of the top-5 markers for (**A**) lymphoid and (**B**) myeloid subclusters. UMAP plots were cropped from the whole LG atlas to visualize the repartition of the subclusters. Clusters are colored by identity. The color gradient on the heatmap corresponds to the scaled average expression level (red: high; blue: low) and the upper bar is colored by cluster identity.

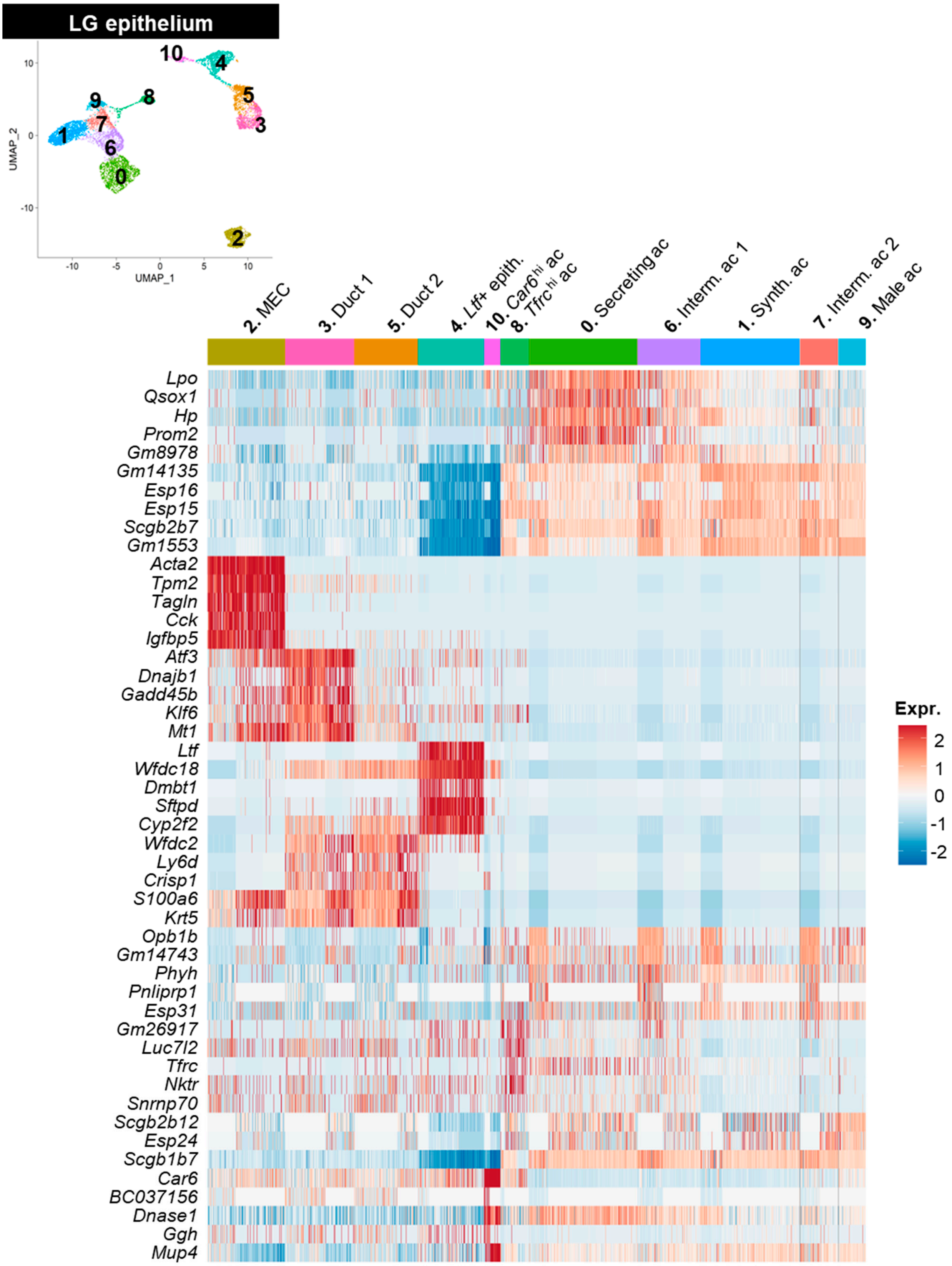

Figure S5. Clustering analysis of the epithelial compartment of the mouse lacrimal gland. The epithelial clusters identified on the whole LG atlas were subjected to a separate analysis to identify

epithelial subclusters (see UMAP plot above). Gene expression heatmap shows the top-5 markers for all epithelial subclusters. The color gradient on the heatmap corresponds to the scaled average expression level (red: high; blue: low) and the upper bar is colored by cluster identity.

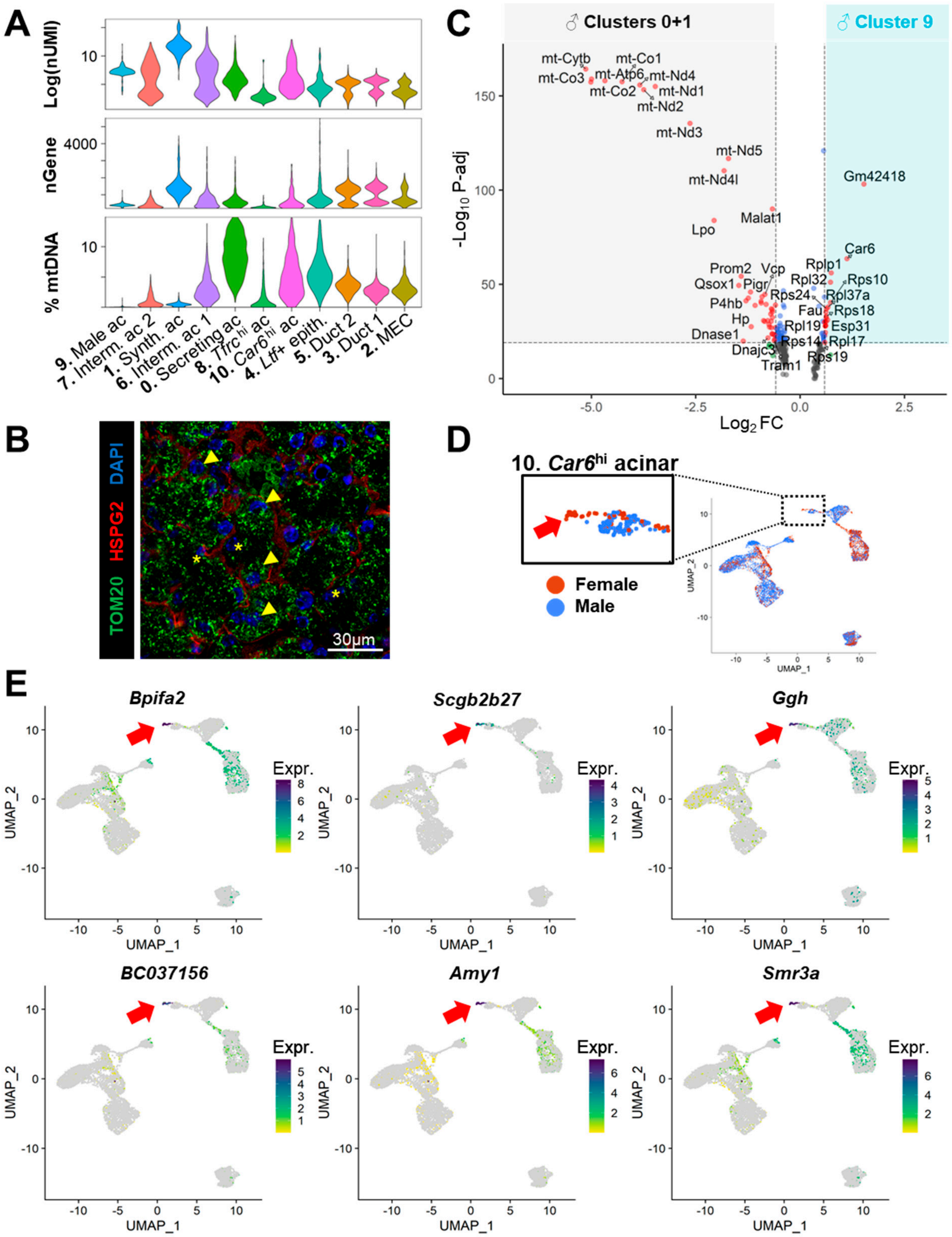

Figure S6. Acinar clusters of the mouse lacrimal gland.
(**A**) Distribution of the number of RNA molecules (nUMI, log scale), genes (nGene) and percent of mitochondrial genes (%mtDNA) across clusters of the LG epithelium. (**B**) Confocal image of a 2

months old LG frozen section immunostained for the mitochondrial marker TOM20 (green) and heparan sulfate proteoglycan (HSPG2, red) that labels the basement membrane. Nuclei were counterstained with DAPI. Arrowheads indicate acinar cells harboring numerous mitochondria, while asterisks indicate acinar cells with low TOM20 staining, suggesting a reduced mitochondrial content. (**C**) Volcano plot for differentially expressed genes between male acinar cluster #9 and male acinar clusters #0,1. Only genes expressed in at least 20% cells of either of the two clusters were considered. Dashed lines intersect the x-axis at fold-change = ± 1.5 and the y-axis at p-adj = 10e-20. Positive fold-changes indicate an upregulation in male cluster #9 compared to male clusters #0,1. (**D**) The small cell subset of the *Car6*^{hi} cluster that differs in expression of multiple genes (red arrow) belongs to the female sample. (**E**) UMAP plot for genes expressed at high levels specifically in the female *Car6*^{hi} subset of cells (red arrow). Cells are colored based on their expression level for the corresponding gene (blue is high). Cells with no expression are shown in grey.

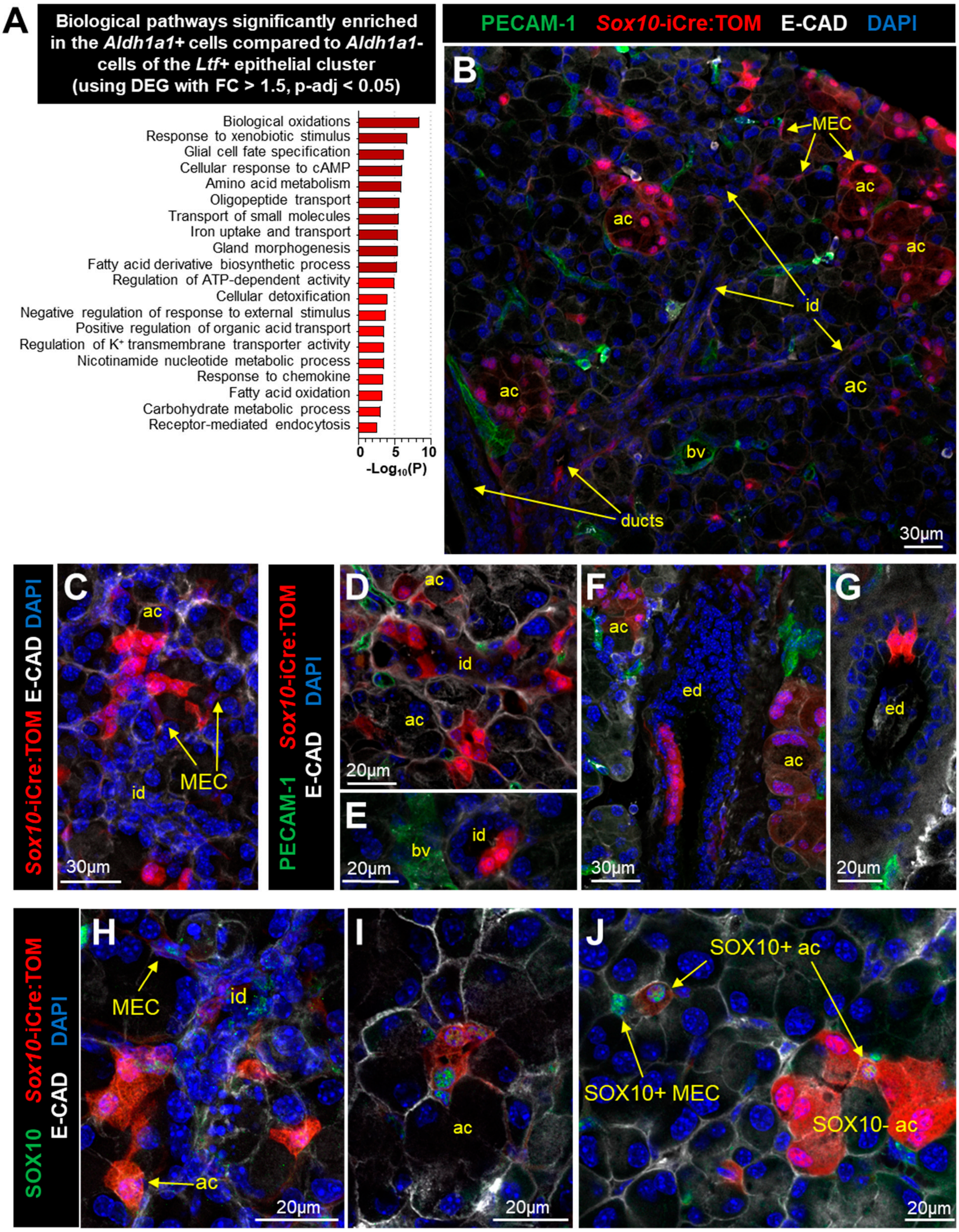

Figure S7. Analysis of putative progenitors of the mouse lacrimal gland.

(**A**) Pathway enrichment analysis for the list of 77 genes significantly upregulated (FC > 1.5, p-adj < 0.05) in *Adh1a1*+ cells compared to *Adh1a1*- cells in the *Ltf*+ cluster. Pathway enrichment analysis was done with Metascape (metascape.org) using default parameters and the following databases: GeneOntology (Biological process), WikiPathways and Reactome. Significant terms were ordered according to their enrichment p-values. (**B-J**) Representative confocal images of LG frozen sections from the *Sox10*-iCre:TOM^{fl} reporter mouse (2 months old) injected with tamoxifen (TM) for two days

and sacrificed one week later. Sections were stained for the epithelial marker E-cadherin (E-CAD, white) and (**B, D-G**) endothelial marker PECAM-1 (green), or (**H-J**) SOX10 antibody. (**B-G**) Epithelial cells derived from *Sox10*-expressing cells at the time of TM-injection are labeled by tdTomato (TOM) and are mainly acinar (ac), myoepithelial (MEC), intercalated duct (id) cells. Less frequently, they are also found in excretory ducts (ed). (**H-J**) Most cells harboring SOX10+ nuclear staining at the time of sacrifice are MECs and cells located in intercalated duct cells and at the junction with acini, or in the vicinity of acini lumen. Large TOM-labeled acinar cells do not show SOX10 nuclear staining, while SOX10+ acinar cells are smaller, either isolated or close to clusters of SOX10-/TOM+ large acinar cells.

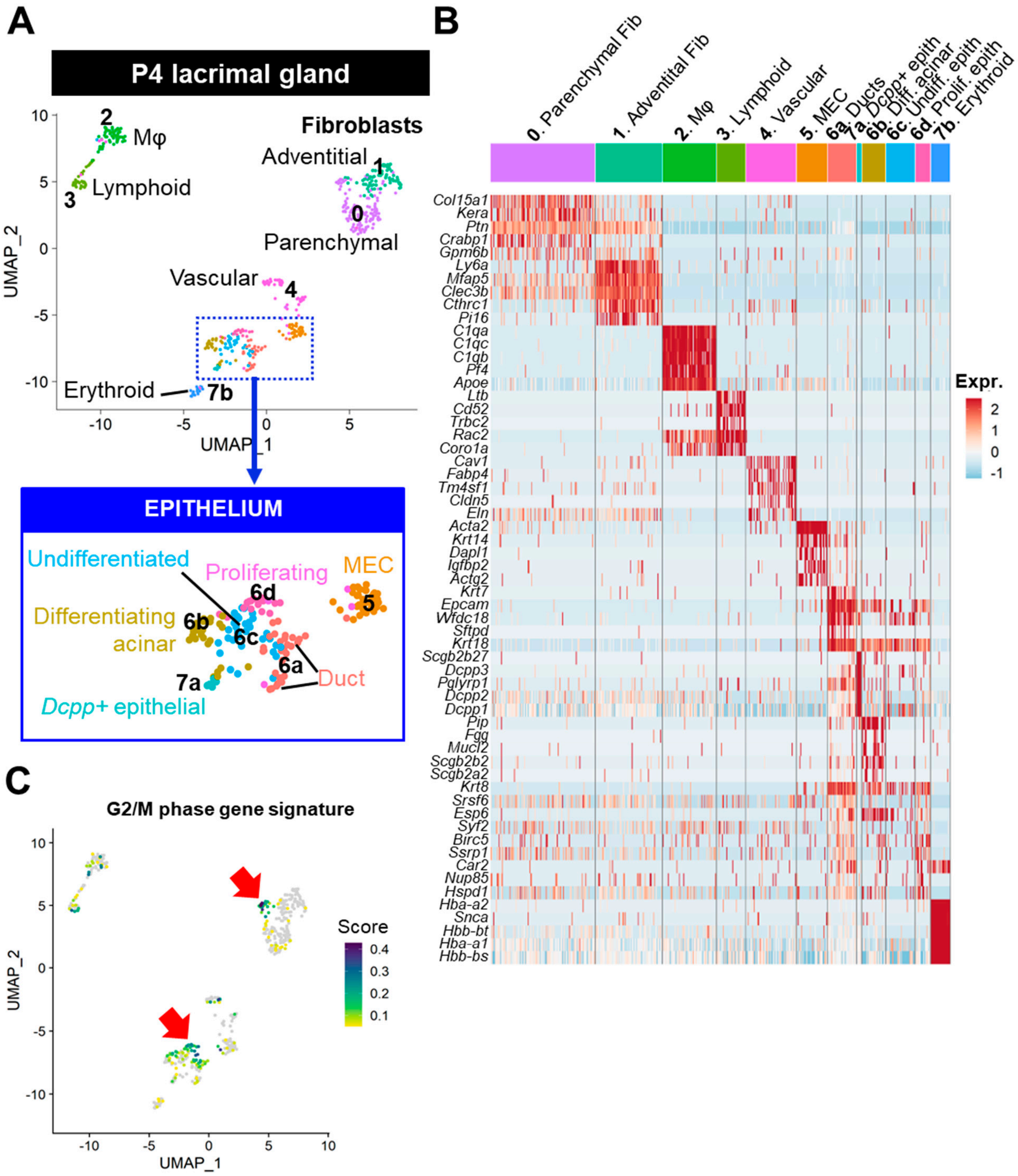

Figure S8. Analysis of the mouse lacrimal gland epithelium at postnatal day 4 (P4).
(**A**) UMAP plot of the LG at P4. Frame with dotted lines delineates the epithelial fraction that is shown below at higher magnification to better visualize epithelial subclusters. (**B**) Gene expression heatmaps of the top-5 markers for the clusters identified in the P4 LG. Clusters are colored by identity. The color gradient on the heatmap corresponds to the scaled average expression level (red: high; blue: low). (**C**) UMAP plot showing cell scores for the signature of genes involved in the G2/M phase of the cell cycle as determined by the R package UCell. Highest scores (blue) are found in clusters of fibroblasts and epithelial cells indicated by red arrows.

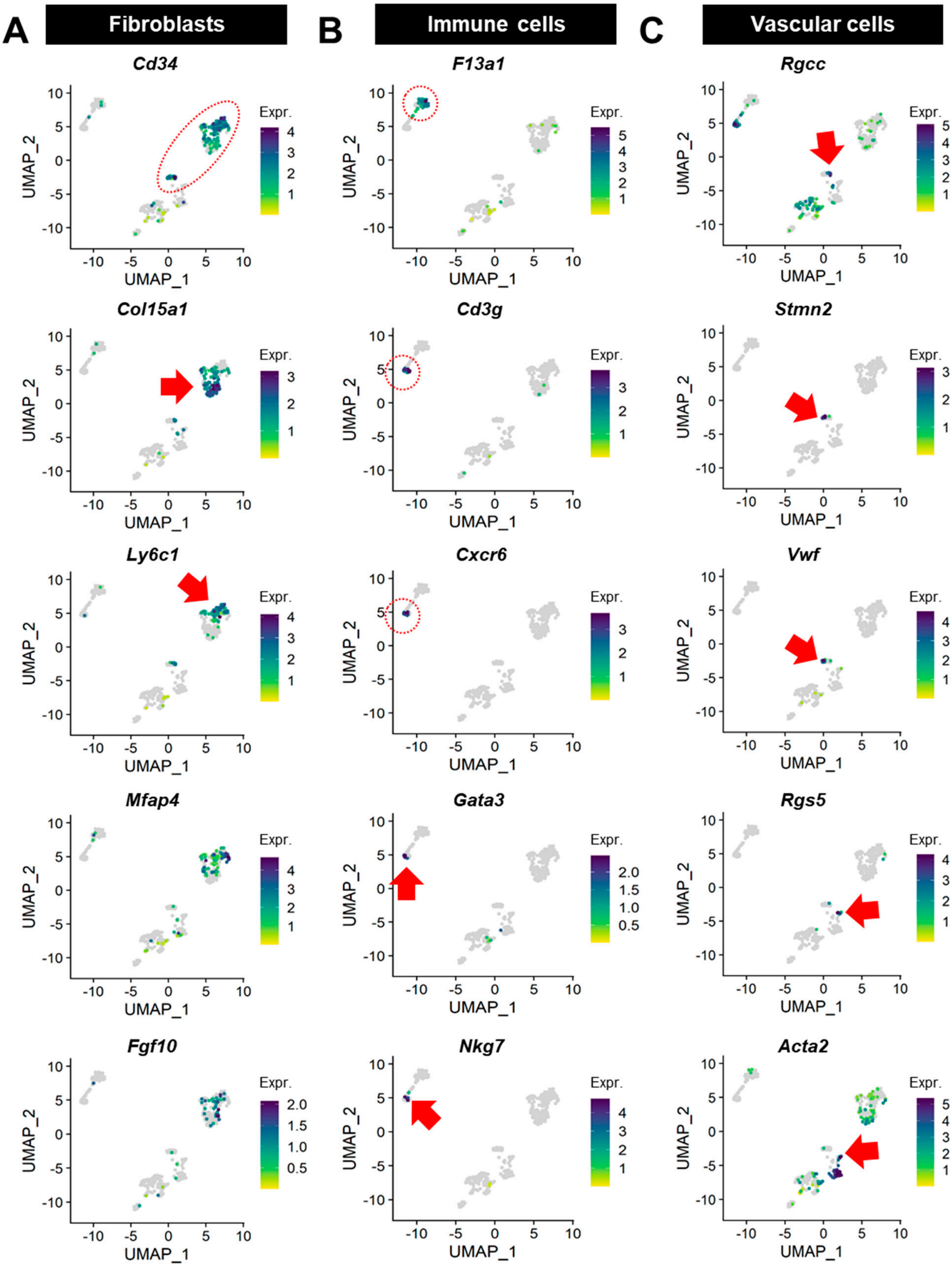

Figure S9. The LG at P4 recapitulates the stromal populations present in the adult LG.
UMAP plots of the P4 LG showing the normalized expression of key genes identified in the two months old mouse LG in the (**A**) fibroblastic, (**B**) immune and (**C**) vascular compartments. Cells are colored based on their expression level for the corresponding gene (blue is high). Cells with no expression are shown in grey. (**A**) *Cd34* expression suggests a progenitor-like phenotype for

mesenchymal cells, including *Col15a1*+ parenchymal-like and *Ly6c1*+ adventitial-like fibroblasts. *Mfap4* was broadly detected, suggesting it is expressed by immature fibroblasts. At this stage, *Fgf10*+ fibroblasts were already detected. (**B**) The immune compartment contained *F13a1*+ resident macrophages and cells of the lymphoid lineage that consisted of resident T cells (*Cd3g, Cxcr6*), and possibly ILC (*Gata3*) as well as NK cells (*Nkg7*). (**C**) In the vascular compartment, similar to adult LG, we also found cells positive for genes expressed by capillaries (*Rgcc*), arterioles (*Stmn2*), venular cells (*Vwf*), and pericytes (*Rgs5, Acta2*).

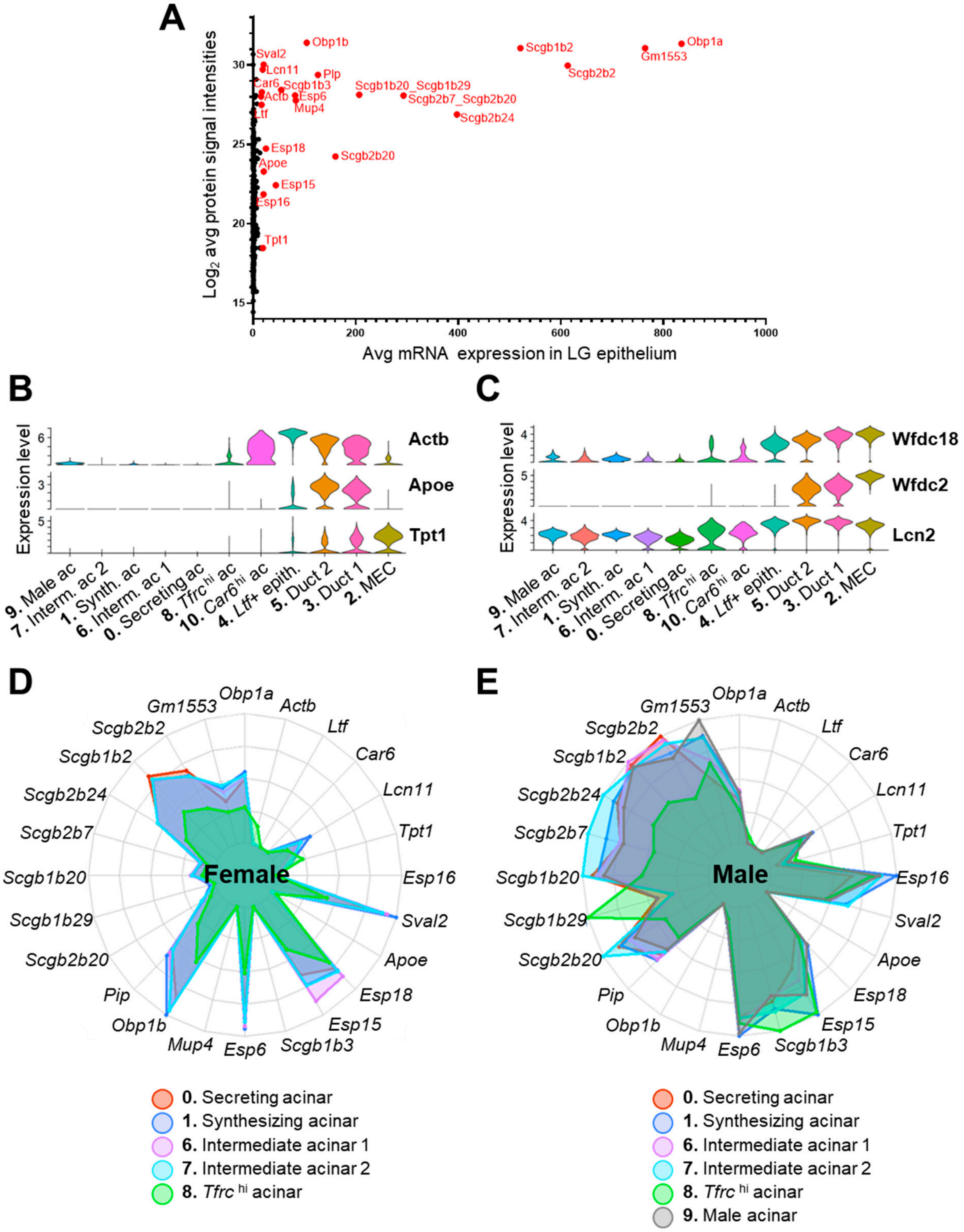

Figure S10. Analysis of the secretome of epithelial LG cells in the light of tear proteome data. (**A**) Average expression level of tear components in the LG epithelium and their corresponding signal intensities by LC-MS/MS analysis of mouse tears. Twenty-three highly abundant tear proteins (in red) were retained for further analysis. (**B-C**) Expression level of tear components expressed at higher levels by other epithelial cells than acinar clusters. Violins are colored by cluster identity. (**D-E**) Spider charts summarizing the average level of tear components in (**D**) acinar clusters #0,1,6,7,8 of the female LG and (**E**) acinar clusters #0,1,6,7,8,9 of the male LG. Webs are colored according to the cluster they correspond to. For each transcript, the maximum of the chart corresponds to the

maximal average expression across clusters for the corresponding gene. While differences in the secretome are obvious between males and females, acinar clusters have similar secretory profiles at the transcriptional level within one sample type.

Description of supplementary tables

Table S1, relative to Figure 1 (separate file)

Table of the top-25 markers for the main clusters of the mouse lacrimal gland analyzed at 2 months old. The first sheet contains the cluster markers generated using the *FindAllMarkers* function of Seurat, with genes expressed in at least 25% of cells in the cluster of interest and a fold-change (FC) > 1.25. The second sheet was generated using the *FindConservedMarkers* function from Seurat to identify the markers the most conserved across samples.

Table S2, related to Figure 4 (separate file)

Table of the top-25 cluster markers for mouse LG epithelium subclustering. Marker genes were generated using the *FindAllMarkers* function of Seurat: only genes expressed in > 25% of cells in the corresponding cluster and with FC > 1.25 were considered.

Table S3, related to Figures 5 and 7B (separate file)

Differential expression analysis between epithelial clusters. Differentially expressed genes (DEGs) were computed using the *FindMarkers* function in Seurat if they passed the following threshold: expression in at least 20% of cells in either one of each cluster and log2(FC) = ±1.25.
The first sheet is the comparison of acinar cluster #0 with acinar cluster #1. The second sheet compares male acinar cluster #9 to male acinar clusters #0-1. The third sheet is the comparison of ductal cluster #3 to ductal cluster #5.

Table S4, related to Figures 8 and S7A (separate file)

Differential expression analysis between *Aldh1a1*+ cells and *Aldh1a1*- cells within the *Ltf*+ epithelial cluster #4. Differentially expressed genes (DEGs) were computed using the *FindMarkers* function in Seurat if they passed the following threshold: expression in at least 20% of cells in either one of each cluster and log2(FC) = ±1.25. DEGs with FC > 1.5 and p-adj <0.05 (in red) were submitted to Metascape (metascape.org) for the pathway enrichment analysis presented in **Fig. S7A**.

Table S5, related to Figures S9 and S8A (separate file)

Table of the top-25 cluster markers for mouse LG at postnatal day 4. Marker genes were generated using the *FindAllMarkers* function of Seurat: only genes expressed in > 25% of cells in the corresponding cluster and with FC > 1.25 were considered.

Table S6, related to Figures 10 and S10 (separate file)

Data of the tear proteome and corresponding mRNA expression in the LG epithelium as evaluated by scRNAseq. The first sheet shows the entire data available about the tear proteome previously published by Stopkova *et al* (2017), and the corresponding genes with their respective average expression in the whole LG epithelium. Genes not detected by scRNAseq are indicated by "N/A". For proteomic data where two proteins are combined, the average gene expression level is the sum of the average expression of each of these genes. The second sheet shows the average protein and mRNA abundance regarding the selected 23 tear components highly expressed in the LG epithelium (in each cluster and in the acinar compartment presented in **Fig. 10**). Differences between males and females are shown with the FC and corresponding p-values.
